# Speech perception under adverse conditions: insights from behavioral, computational, and neuroscience research

**DOI:** 10.3389/fnsys.2013.00126

**Published:** 2014-01-03

**Authors:** Sara Guediche, Sheila E. Blumstein, Julie A. Fiez, Lori L. Holt

**Affiliations:** ^1^Department of Cognitive, Linguistic, and Psychological Sciences, Brown UniversityProvidence, RI, USA; ^2^Department of Cognitive, Linguistic, and Psychological Sciences, Brain Institute, Brown UniversityProvidence, RI, USA; ^3^Department of Neuroscience, Center for Neuroscience at the University of Pittsburgh, University of PittsburghPittsburgh, PA, USA; ^4^Department of Psychology, University of PittsburghPittsburgh, PA, USA; ^5^Department of Psychology at Carnegie Mellon University and Department of Neuroscience at the University of Pittsburgh, Center for the Neural Basis of CognitionPittsburgh, PA, USA; ^6^Department of Psychology, Carnegie Mellon UniversityPittsburgh, PA, USA

**Keywords:** perceptual learning, plasticity, supervised learning, cerebellum, language, prediction error signals, speech perception

## Abstract

Adult speech perception reflects the long-term regularities of the native language, but it is also flexible such that it accommodates and adapts to adverse listening conditions and short-term deviations from native-language norms. The purpose of this article is to examine how the broader neuroscience literature can inform and advance research efforts in understanding the neural basis of flexibility and adaptive plasticity in speech perception. Specifically, we highlight the potential role of learning algorithms that rely on prediction error signals and discuss specific neural structures that are likely to contribute to such learning. To this end, we review behavioral studies, computational accounts, and neuroimaging findings related to adaptive plasticity in speech perception. Already, a few studies have alluded to a potential role of these mechanisms in adaptive plasticity in speech perception. Furthermore, we consider research topics in neuroscience that offer insight into how perception can be adaptively tuned to short-term deviations while balancing the need to maintain stability in the perception of learned long-term regularities. Consideration of the application and limitations of these algorithms in characterizing flexible speech perception under adverse conditions promises to inform theoretical models of speech.

Spoken language is conveyed by transient acoustic signals with complex and variable structure. Ultimately, the challenge of speech perception is to map these signals to representations (e.g., pre-lexical and lexical knowledge) of an individual's native language community. In real-world environments, this challenge is frequently exacerbated under adverse listening conditions arising from noisy listening environments, hearing impairment, or speech that deviates from long-term speech regularities due to talkers' accents, dialects or speech disorders. In circumstances where adverse conditions lead to systematic short-term deviations from the long-term regularities of a language, a listener can rapidly adjust the mappings from acoustic input to long-term knowledge. However, little is known about the mechanisms underlying adaptive plasticity in speech perception. Understanding such rapid adaptive plasticity may provide insight into how the perceptual system deals with adverse listening situations. Although there has been recent interest in investigating adaptive plasticity in speech perception, these studies have used different tasks and methodologies and remain mostly unconnected. It is one of the goals of this paper to review these findings and integrate the results within a potentially common framework.

To this end, we examine a number of factors that influence adaptive plasticity in speech perception and review behavioral, computational, and functional neuroimaging studies that have contributed to our current understanding of adaptive processes. In reviewing these mostly separate strands of research, we take the view that examining candidate neural systems that may underlie the behavioral changes could reveal a unifying framework for understanding how adaptive plasticity is achieved. We draw from domains outside of speech perception to consider supervised learning relying on sensory prediction error signals as a potential mechanism for uniting seemingly distinct behavioral speech perception phenomena. From this perspective, we propose that understanding the neural basis of adaptive plasticity in speech perception will require integrating subcortical structures into current frameworks of speech processing, which until now have largely focused on the cerebral cortex. Specifically, we examine the possibility that subcortical-cortical interactions may form functional networks for driving plasticity.

## Insights from behavioral studies

We first examine two distinct behavioral literatures, each demonstrating adaptive changes in speech perception in response to signal distortions. One set of studies investigates improvements in *spoken word recognition* following experience with distorted signals. The other examines changes in *acoustic phonetic perception* following experience with distorted input in disambiguating contexts. Both sets of studies show changes at early stages of speech processing, which are facilitated by disambiguating contextual sources of information (e.g., lexical information). Across different studies and tasks, perceptual effects showing adaptive changes in speech perception have been variously termed “perceptual learning,” “adaptation,” “recalibration,” and “retuning,” with the choice of descriptor driven mostly by the associated task. Here, we use “adaptive plasticity” as a broader term to be inclusive of distinct literatures and different tasks that may tap into some of the same processes in adjusting speech perception to accommodate short-term deviations in speech acoustics.

### Adaptive plasticity in word recognition tasks

Adults rapidly and effortlessly extract words from fluent speech. However, adverse listening conditions can affect the quality and reliability of the acoustic speech signal and negatively impact word recognition, reducing intelligibility (for review see Mattys et al., 2012). Under certain circumstances, brief experience with the adverse listening condition results in intelligibility improvements (e.g., Pallier et al., 1998; Liss et al., 2002; Clarke and Garrett, 2004; Bradlow and Bent, 2008). For example, several studies have shown that brief familiarization with natural foreign-accented speech can improve intelligibility of the accented talker (e.g., Clarke and Garrett, 2004; Bradlow and Bent, 2008) and, under some circumstances, generalize to intelligibility improvements for speech from other talkers with the same native language background (e.g., Bradlow and Bent, 2008). Such adaptive plasticity is observed across many acoustic speech signal distortions including synthesized text-to-speech (Schwab et al., 1985; Greenspan et al., 1988; Francis et al., 2000) dysarthric speech (Liss et al., 2002), and speech in noise (e.g., Cainer et al., 2008). It is also observed with more synthetic manipulations of the speech signal such as noise vocoding (Davis et al., 2005), spectral shifting (e.g., Fu and Galvin, 2003), and time compression (e.g., Altmann and Young, 1993). Many of these experimental manipulations relate to commonly occurring natural adverse listening experiences and some are intended to mimic the degraded experiences encountered by listeners with hearing deficits or cochlear implants. Overall, there is widespread evidence that intelligibility of distorted speech input improves with relatively brief experience or training across many different types of signal distortion.

Though the flexibility of perception under a variety of adverse listening conditions indicates the robustness of adaptive plasticity in speech perception, the use of different stimulus manipulations and different types of training and experience across studies makes it difficult to build an integrative model. However, several key characteristics merit special attention. One significant characteristic of studies in this literature is the supportive influence of information that disambiguates the acoustics of distorted words. This information may originate from external feedback indicating the appropriate interpretation of the signal. For example, intelligibility is improved when a distorted acoustic word is paired with the written form of the word during the initial presentation (e.g., Fu and Galvin, 2003) or following the response (e.g., Greenspan et al., 1988; Francis et al., 2000, 2007), and when the clear undistorted version of the signal precedes the distorted signal during training (Hervais-Adelman et al., 2008). Each of these approaches provides the speech system with information to support mapping the distorted speech signal to linguistic knowledge.

Adaptive plasticity in speech perception can even occur without explicit feedback. Mere exposure to nonnative-accented speech results in improvements in performance in the absence of explicit feedback or other explicit information about the correct interpretation (e.g., Altmann and Young, 1993; Mehler et al., 1993; Sebastian-Galles et al., 2000; Liss et al., 2002). Simply listening to time-compressed speech (Altmann and Young, 1993; Mehler et al., 1993; Sebastian-Galles et al., 2000) or natural speech from dysarthric patients (Liss et al., 2002) can lead to intelligibility improvements. Likewise, experience with distorted sentences containing real target words improves recognition of subsequent distorted sentences to a greater degree than experience with target nonwords (Davis et al., 2005). These findings suggest that internally generated lexical information may also contribute to adaptive plasticity. In sum, information that supports the disambiguation of speech, including externally provided information and internally generated lexical information, may promote adaptive plasticity (Davis et al., 2005; Hervais-Adelman et al., 2008).

A second significant characteristic of adaptive plasticity is that when external sources of information are unavailable to resolve the ambiguity of the distorted acoustic signal, the degree of adaptation appears to be dependent on the severity of the distortion (Bradlow and Bent, 2008; Li et al., 2009). For example, listeners show greater adaptation to relatively more intelligible foreign accented speech (Bradlow and Bent, 2008). Other studies have shown that adaptive plasticity is difficult for severe artificial speech distortions (e.g., Li and Fu, 2006, 2010), whereas gradually increasing the severity of the distortion (Guediche et al., 2009) or intermixing less severely distorted signals with more severe distortions (Li et al., 2009) facilitates adaptation. Indeed, adaptive plasticity can be readily observed for time-compressed speech, even without external feedback (e.g., Pallier et al., 1998). This may be because the degree of time compression generally used tends to result in more intelligible distortions (often 50–60% intelligibility or greater) (e.g., Pallier et al., 1998; Peelle and Wingfield, 2005; Adank and Janse, 2009) in comparison to text-to-speech or noise-vocoded speech distortions (e.g., Schwab et al., 1985; Francis et al., 2000; Davis et al., 2005; Hervais-Adelman et al., 2011) that typically employ feedback to promote adaptive plasticity.

A third key characteristic of adaptive plasticity is that improvements in intelligibility as a consequence of experience generalize to words not encountered during a training or exposure period (Schwab et al., 1985; Francis et al., 2000, 2007). In fact, in many studies all the words in the experiment are unique. Therefore, even though lexical knowledge can mediate adaptive plasticity by disambiguating the distorted signals (Davis et al., 2005), adaptive change must occur in the mapping of the distorted sounds to pre-lexical representations and not in the mapping from speech acoustics to any particular lexical item.

Overall, studies of adaptive plasticity in word recognition employ multiple stimulus distortions and various approaches to delivering experience with the speech distortion. Experience with the speech distortions can lead to improvements in intelligibility, through adaptive processes that retune the mapping of the distorted acoustic speech input to the speech processing system. The remapping seemingly plays out at an early stage of perception (e.g., pre-lexical). This adaptive plasticity is facilitated by the availability of disambiguating external information (such as explicit feedback or corresponding clear and undistorted speech), and also by signals that are relatively less distorted and, therefore, more intelligible. Disambiguating information and baseline intelligibility may have their influence on adaptive plasticity through a common means: each may impact the relative accuracy with which the distorted acoustics are mapped to established long-term regularities of the native language.

If both externally-provided and internally-generated information contribute to adaptive plasticity, the impact of external feedback on adaptive plasticity is likely to be greater for less intelligible signal distortions compared to more intelligible distortions. Indeed, when distortion intelligibility and the presence of external feedback are independently manipulated, the two factors interact to modulate the degree of adaptive plasticity observed (Guediche et al., 2009). Intelligibility serves as a metric for the accuracy with which listeners can map distorted signals to lexical knowledge. Greater intelligibility thus indicates greater success in mapping distorted acoustics, which may produce internal signals to guide adaptive plasticity that are less reliable or less available when intelligibility is low. In this latter case, external information that supports accurate mapping may serve to drive adaptive plasticity. We return to the implications of this possibility below.

### Adaptive plasticity in acoustic phonetic perception

Adaptive plasticity has also been shown in other speech tasks that examine acoustic phonetic perception. Acoustic phonetic perception involves a complex mapping of acoustic speech signals that vary along multiple, largely continuous acoustic dimensions to long-term representations that respect the regularities of the native language (e.g., phonemes, words). This mapping is complicated by the fact that even when measured in quiet, well-controlled laboratory conditions, the acoustics conveying a particular phoneme or word are highly variable (e.g., Peterson and Barney, 1952).

Under adverse conditions more typical of natural listening environments, there are short-term deviations in speech acoustics introduced by sources like foreign accent, dialect, noise, different speakers, and speech disorder. These systematic deviations can distort the acoustic speech signal. A listener may encounter a native Spanish talker referring in English to a *fish* using a vowel with acoustics more typical of English /i/ (a *feesh*) than /I/. The same listener might also encounter a native Pittsburgh talker chatting about the local football team, the Steelers, in the local dialect that produces English /i/ with acoustics more typical of /I/ (the *Stillers*). Listeners would have little difficulty in either case as the perceptual system flexibly adjusts to such signal distortions.

A broad research literature with a long history demonstrates that ambiguous speech signals can be resolved using many sources of contextual information. Acoustic (Lotto and Kluender, 1998; Holt, 2005), lexical (Ganong, 1980), visual (McGurk and Macdonald, 1976; MacDonald and McGurk, 1978), and sentence contexts (Ladefoged and Broadbent, 1957), among others, each play a role in disambiguating speech signals. A sound with ambiguous acoustics between /g/ and /k/ is more likely to be perceived as /k/ in the context of __*iss* (*kiss* is a real word, *giss* is not), but as /g/ in the context of __*ift* (Ganong, 1980). Similarly, an ambiguous sound between /b/ and /d/ can be disambiguated by watching a video of a face articulating /b/ vs. /d/ (Bertelson et al., 1997). Relevant to the adaptive plasticity literature, repeated exposure to an ambiguous acoustic speech signal in a disambiguating context affects later perception of the ambiguous speech—even in the absence of a biasing context (Norris et al., 2003; Vroomen et al., 2007). This suggests an adaptive change in the way the ambiguous speech acoustics are mapped that remains even when the biasing context is no longer available.

Two such biasing contexts have been explored extensively, lexical context and visually-presented articulating faces (Bertelson et al., 2003; Norris et al., 2003; Vroomen et al., 2007). Lexically-mediated changes in acoustic phonetic perception can be achieved by exposing listeners to ambiguous speech sounds embedded in lexical contexts that only produce a valid lexical item for one of the phonemes (e.g., Norris et al., 2003; Kraljic and Samuel, 2005; Maye et al., 2008; for review see Samuel and Kraljic, 2009 for review). For example, when an acoustically-ambiguous sound between /s/ and [∫] is presented in contexts for which only /s/ completes a real word (e.g., *legacy, Arkansas*), lexical knowledge provides a means of disambiguating the sound (Ganong, 1980). This experience affects subsequent [s]-[∫] perception such that the acoustically-ambiguous [s]-[∫] sound is more broadly accepted as [s] following exposure to [s]-consistent lexical contexts than following exposure to [∫] -consistent contexts (e.g., *pediatrician;* Kraljic and Samuel, 2005). This effect is observed even when the lexically-biasing context is no longer present. Many experiments have demonstrated such lexical tuning of acoustic phonetic perception across phonemes, languages, and talkers in adults (see for review Samuel and Kraljic, 2009) and even among 6- and 12-year-old children (McQueen et al., 2012).

Exposure to visual information from an articulating face that disambiguates an ambiguous speech sound produces similar changes in acoustic phonetic perception. Bertelson et al. (2003) examined phonetic perception of an acoustically-ambiguous /aba/ and /ada/. Following exposure to the ambiguous token paired with a video of a face clearly articulating /aba/, subsequent perception of the ambiguous /aba/-/ada/ stimuli was shifted as acoustic information consistent with /aba/.

Although lexical and visually-mediated adaptive plasticity have been most studied to date, other factors can also drive adaptive plasticity. Phonotactic probabilities (Cutler, 2008) and statistical regularities experienced across multiple tokens of speech exemplars (Clayards et al., 2008; Idemaru and Holt, 2011) can also result in adaptive plasticity. In the latter example, correlations among acoustic cues provide a disambiguating source of information for how acoustic dimensions relate to one another in signaling phonemes (Idemaru and Holt, 2011). These findings are consistent with a rich literature demonstrating that listeners make use of many sources of information to disambiguate inherently ambiguous acoustic speech input. The literature on adaptive plasticity extends these observations by demonstrating that upon repeated exposure, the effects of a disambiguating context can remain even in the absence of context.

Clarke-Davidson et al. (2008) argue that data demonstrating adaptive plasticity in acoustic phonetic perception are best fit by modeling adaptation at the level of perceptual (pre-lexical) processing rather than at a subsequent decision level. In general, the nature of this pre-lexical influence is to more broadly accept the ambiguous acoustics as consistent with the biasing context. In other words, the adaptive adjustments of acoustic-phonetic perception are in the direction of the disambiguating (lexical, visual, statistical) contexts. In this way, adaptive plasticity in acoustic phonetic perception bears resemblance to adaptive plasticity in word recognition reviewed above. Specifically, both examples of adaptive plasticity show that contextual information (e.g., lexical information) can drive changes in perception at a pre-lexical level.

### Summary

Two largely independent strands of research demonstrate rapid adaptive changes in the mapping of distorted acoustic speech signals. They have evolved in parallel, kept distinct primarily along paradigmatic lines, with little cross-talk (although Norris et al. (2003), Cutler (2008), and Samuel (2011) note commonalities). Motivated by results across these studies that show similarities, such as the contributions of both internal (e.g., lexical) and external (e.g., feedback) information sources, a common pre-lexical locus, and a similar influence of the severity of the acoustic distortion on the degree of adaptation, we explore the possibility that these commonalities reflect common mechanisms. We first review computational modeling efforts that account for adaptive plasticity, and then turn to cognitive neuroscience and neuroscience research in other domains for further insights.

## Insights from computational modeling

Computational models assist in understanding adaptive plasticity by explicitly modeling outcomes of potential learning algorithms and relating these outcomes directly to behavioral evidence. Traditional computational models of speech perception are generally defined by hierarchically-organized layers that represent linguistic information at different levels of abstraction (e.g., perceptual/featural, pre-lexical, lexical). Two classes of hierarchical models—feedforward models (e.g., Norris, 1994; Norris et al., 2000) and interactive models (e.g., McClelland and Elman, 1986; Gaskell and Marslen-Wilson, 1997)—have been especially influential and each has provided an account of rapid adaptive plasticity, specifically focusing on lexically-mediated adaptive plasticity as measured by changes in acoustic phonetic perception (e.g., Norris et al., 2003). In the interactive model Hebb-TRACE, an unsupervised learning algorithm, Hebbian learning, is used to modify connection weights (Mirman et al., 2006), whereas a supervised learning algorithm (backpropagation) is proposed in the context of the feedforward MERGE model (Norris et al., 2003).

One influential debate between feedforward and interactive accounts is the degree to which different levels interact with one another. In feedforward modes like MERGE, there is no direct feedback from lexical representations to influence online speech perception. Thus, in contrast to interactive models, adaptive plasticity arises from feedback that is dedicated only for the purpose of learning. Norris et al. (2003) propose that in this case, feedback from lexical to pre-lexical levels is used to derive an error signal that indicates the degree to which there is a discrepancy between the expected phonological representation activated by the lexical item and the one indicated by the acoustic speech signal. They propose backpropagation, first instantiated by Rumelhart et al. (1986), as an implementation of supervised learning to produce adaptive plasticity. Backpropagation uses error signals to drive changes in the weights of connections between the input speech signal and the pre-lexical information to reduce the discrepancy. Because the pre-lexical units mediate mapping between acoustic input and lexical knowledge, generalization to new words also results. While backpropagation provides a supervised learning mechanism that may capture the rapid nature of the observed behavioral effects, it is not neurobiologically plausible (Crick, 1989).

Hebb-TRACE (Mirman et al., 2006) is a modification of the interactive TRACE model (McClelland and Elman, 1986) that has an added Hebbian learning algorithm. It models adaptive plasticity via adjustments in the weights mapping from input to pre-lexical representations. Lexical activation results in direct excitatory feedback from the lexical layer to pre-lexical information consistent with the word. Processing of a perceptually ambiguous sound (e.g., with acoustics between /s/ and /∫/) leads to partial activation of both consonants with lateral inhibitory within-level connections leading to competition between the two alternatives at the pre-lexical level. The biasing lexical context (e.g., *legacy, Arkansas*) increases the activation of the congruent phoneme (/s/) through direct excitatory feedback, granting it an advantage over the partially-activated /∫/. To achieve adaptive plasticity, the mapping of lower-level perceptual information to phonetic categories is adjusted via Hebbian learning such that subsequent perception of these consonants is more likely to activate the consonant consistent with the previous lexical context, even in the absence of the biasing context. By this account, the same lexical feedback that influences online acoustic phonetic perception also guides learning of the mapping of distorted speech onto pre-lexical representations. A difficulty for this account is its time course. Whereas adaptive plasticity effects can require as few as 10–20 trials to evoke, Hebbian learning has a much slower time course for learning (Norris et al., 2003; Vroomen et al., 2007).

Although the focus of traditional computational accounts has been on modeling the effect of lexical information on acoustic phonetic perception, the proposed learning mechanisms may be capable of accounting for adaptation to distorted speech input of the sort observed in the word recognition literature. Norris et al. (2003) explicitly make the connection between the mechanisms involved in adaptive plasticity of acoustic phonetic perception and those that underlie improvements in word recognition. The proposed mechanisms for lexically-guided adaptive plasticity in both MERGE and Hebb-TRACE also could be extended to accounts of other types of lexically-mediated adaptive plasticity and effects of other linguistic information at other higher levels of linguistic abstraction [e.g., sentence context (Borsky et al., 1998)], or different modalities (e.g., visual information Vroomen et al., 2007). Nonetheless, to this point, these disparate strands of research have not been integrated and there have been few attempts to examine whether it may be possible to unite different phenomena of adaptive plasticity in speech perception on mechanistic grounds.

### A unifying perspective?

The behavioral and modeling literatures that investigate adaptive plasticity in speech processing have distinct approaches that make it challenging to draw direct comparisons. However, evaluating them together reveals that there are a few observations any account of adaptive plasticity must address. One is that information that disambiguates distorted or otherwise perceptually-ambiguous acoustic speech input rapidly adjusts the way that the system maps speech input at a pre-lexical level, such that later input is less ambiguous even when disambiguating information is no longer present to support interpretation. Long-term knowledge, external feedback, and overall intelligibility of the distorted input each seem to play a role in modulating the extent to which adaptive plasticity is observed.

A common feature among different forms of disambiguating information may be that they each provide a basis for generating predictions. This characteristic relates to recent work suggesting that predictive coding may be a useful framework for understanding speech processing. To this end, we use predictive coding as an illustrative approach for considering adaptive plasticity. Predictive coding models capitalize on the reciprocal connections between different levels of a hierarchically organized structure and provide a way for generating predictions from externally-provided context or from internally-accessed information induced by the stimulus itself (Bastos et al., 2012; Panichello et al., 2012). The idea is that feedback from higher levels in the hierarchical speech processing structure can modulate activity in lower levels. These predictions are compared with the actual sensory input such that any discrepancies result in an internally-generated prediction error signal. This error signal, in turn, drives adaptive adjustments of the internal prediction to improve alignment of future predictions with incoming input. Although there is still debate regarding the role of different sources of feedback in online perception compared to adaptive plasticity (Norris et al., 2000; McClelland et al., 2006), the generation of predictions and prediction error signals may be common to both processes.

In the domain of adaptive plasticity for acoustic phonetic perception, Vroomen and colleagues suggested that “crossmodal conflict” is responsible for driving rapid changes in perception and noted the possibility that it provides a common mechanism for both lexically-mediated and visually-mediated adaptive plasticity (Vroomen et al., 2007; Vroomen and Baart, 2012). They argued that in both cases, a discrepancy (i.e., error signal) between the information provided by different sources of information (lexical, visual) and the information provided by the input sensory modality (ambiguous acoustic speech signal) leads to adaptive plasticity. Bertelson et al. (2003); Vroomen et al. (2007), Vroomen and Baart (2012) also noted the intriguing similarities between adaptive plasticity in speech perception and sensorimotor adaptation, such as is observed for adapting movements while wearing visually-distorting prism goggles, Martin et al., 1996b). Namely, each depends on discrepancies between expected and actual sensory outcomes. Although Vroomen et al.'s analogy has been rarely linked to the supervised learning algorithms that are posited as a mechanism of adaptive plasticity in the MERGE model (Norris et al., 2003), it is strikingly similar. Dependence on discrepancies between expectations of the input as a result of lexical activation and the *actual* activation from the input form the basis of prediction error signals of supervised learning for adaptive plasticity and also relate closely to mechanisms attributed to sensorimotor adaptation in literatures outside of speech perception (see Wolpert et al., 2011 for review). Thus, consideration of the mechanisms underlying prediction error signals, generally, and sensorimotor adaptation, more specifically, may reveal a rapid and biologically-plausible neural mechanism for achieving adaptive plasticity in speech perception.

## Insights from cognitive neuroscience

### Neuroimaging evidence for predictive coding in speech perception

Although neuroanatomical models of speech perception differ in their details, the general consensus is that there are two or more hierarchically-organized streams that diverge from posterior superior temporal cortex (Hickok and Poeppel, 2007; Rauschecker, 2011). The popular dual-stream model by Hickok and Poeppel (2007) suggests a ventral stream that supports access to meaning and combinatorial processes, and a dorsal stream that supports access to articulatory processing. In the ventral stream, more posterior areas of temporal cortex are involved in perceptual and lower levels of speech processing, whereas more anterior temporal cortical regions are involved in more abstract higher levels of language processing (Hickok and Poeppel, 2007; Rauschecker and Scott, 2009; DeWitt and Rauschecker, 2012). In particular, superior temporal areas are recruited for sensory-based perceptual processes, posterior middle and inferior temporal areas are engaged in lexical and semantic processes, and anterior superior and middle temporal areas are involved in comprehension (Binder et al., 2004, 2009; Scott, 2012). Supporting evidence for a posterior (responding earlier) to anterior (responding later) ventral processing stream in temporal cortex comes from a variety of neuroimaging methodologies and analyses (e.g., Gow et al., 2008; Leff et al., 2008; Sohoglu et al., 2012). In the dorsal stream, parietal areas have been implicated in sensorimotor processing and frontal areas in articulatory processing. However, there is also evidence for parietal involvement in other aspects of speech processing including semantic and conceptual processes (e.g., Binder et al., 2009; Seghier et al., 2010), lexical and sound categorization (e.g., Blumstein et al., 2005; Rauschecker, 2012). Similarly, other functions have been attributed to frontal areas, such as suggestions that the inferior frontal gyrus (BA44/45) is engaged in syntactic and executive processes (Caplan, 1999, 2006; Binder et al., 2004; Fedorenko et al., 2012). Nonetheless, the view that multiple hierarchically organized neural streams support different aspects of perception has been established as a framework for understanding perception for visual and auditory perception (Ungerleider and Haxby, 1994; Rauschecker and Tian, 2000), and is also becoming a widely accepted view for speech processing (e.g., Hickok and Poeppel, 2004, 2007; Rauschecker and Scott, 2009; Peelle et al., 2010; Price, 2012).

This kind of hierarchically organized system has formed the basis for understanding speech processing. For example, models that propose predictive coding also postulate a system that is hierarchically organized with reciprocal connections between different stages of processing. Although the focus of such models has been on online speech processing rather than adaptive plasticity, understanding how predictions affect changes in brain activity is essential for each of these processes. At the neural level, the predictive coding framework suggests predictions can serve to constrain perception through feedback signals from regions associated with processing information at higher levels of abstraction (e.g., frontal areas that are at higher levels in the speech hierarchy) that modulate activity in regions associated with perceptual processes (e.g., temporal areas that receive the top–down modulation) (for review see Davis and Johnsrude, 2007; Peelle et al., 2010; Wild et al., 2012b). Thus, the literature on predictive coding has focused largely on changes in frontal areas (associated with higher-level processes) and temporal areas (associated with perceptual processes). Based on hypothesized functions of different brain regions, neuroimaging studies have provided some evidence for predictive mechanisms in speech perception (e.g., Clos et al., 2012; Sohoglu et al., 2012; Wild et al., 2012) by examining effects of predictive contexts and stimulus distortions, as well as their interactions.

Consistent with a hierarchically organized predictive coding framework, manipulation of predictive contexts modulates activity in frontal areas, with greater activity typically observed for more predictive contexts (e.g., Myers and Blumstein, 2008; Gow et al., 2008; Davis et al., 2011; Clos et al., 2012; Wild et al., 2012). Not surprisingly, stimulus distortions modulate activity in temporal areas (Davis et al., 2011; Clos et al., 2012; Wild et al., 2012), which are associated with early perceptual processes. Findings from MEG provide supporting evidence that this modulation begins early in the speech processing time course (Sohoglu et al., 2012). Interestingly, effects related to manipulations of speech signal distortion seem to depend on stimulus intelligibility, with greater activity to distortion severity for intelligible stimuli and decreased response to distortion severity for unintelligible stimuli (Poldrack et al., 2001; Adank and Devlin, 2010). This U-shaped response function indicates that modulatory influences of signal distortion in temporal cortex may be dependent on multiple factors. Although not all of the studies examine or report modulatory influences of stimulus distortions on frontal areas, many studies do show increases in frontal activity associated with increases in the distortion severity (e.g., Poldrack et al., 2001; Adank and Devlin, 2010; Eisner et al., 2010).

Since the size of the prediction error signal depends on both the predictive context and the congruency of the acoustic input, one approach has been to examine the interaction between a predictive context and a stimulus distortion in order to determine potential regions that encode error signals (Spratling, 2008; Gagnepain et al., 2012; Clark, 2013). A number of studies have shown such interactions in both temporal and frontal areas (e.g., Obleser and Kotz, 2010; Davis et al., 2011; Obleser and Kotz, 2011; McGettigan et al., 2012; Sohoglu et al., 2012; Guediche et al., 2013). Davis et al. (2011) found an interaction between a semantic coherence manipulation that modulated the degree to which targets were predictable and an acoustic speech signal distortion of those targets in frontal and temporal areas, providing evidence for the involvement of the two regions in predictive coding. Sohoglu et al. (2012) examined the joint effects of the sensory distortion of a spoken word and the informativeness of preceding text resolving the distorted signal, suggesting that both factors modulate activity in temporal cortex albeit in opposing directions. That is, sensory detail evoked greater response, relative alignment of the signal with top-down knowledge resulted in less response. Even more compelling evidence comes from an MEG study that demonstrated changes in activity in the superior temporal gyrus that were modulated based on differences between what was expected and what was heard. This study used a segment prediction error task, in which the beginning segment of a word predicted or did not predict the end segment (formula vs. formubo) (Gagnepain et al., 2012). That temporal areas are involved in early perceptual processes and are also sensitive to this interaction led the authors to conclude that these areas reflect the encoding of prediction errors in speech perception (Clos et al., 2012; Gagnepain et al., 2012; Sohoglu et al., 2012; Wild et al., 2012). Together, the studies suggest that predictive coding, which generates feedback signals (presumably from frontal areas) modulates temporal areas according to the predicted sensory input generated from the predictive coding context.

On the other hand, evidence from other studies suggests that the story may be more complex. For example, across studies, similar manipulations have produced different patterns of changes in BOLD signal (e.g., Davis et al., 2011 vs. Sohoglu et al., 2012). Since changes in BOLD signal may reflect different aspects of the error signal (e.g., degree, precision) (Friston and Kiebel, 2009; Hesselmann et al., 2010), there are still many open questions about the role of different regions in predictive coding. Furthermore, some interactions cannot be completely accounted for by a predictive coding framework (McGettigan et al., 2012; Guediche et al., 2013). In the predictive coding framework, activation within areas reflecting prediction error signals should increase as the degree of discrepancy between the expected and actual input increases. However, some studies have shown interdependent modulatory influences, for example, McGettigan et al. (2012) showed that responses to the *quality* or clarity of the acoustic stimulus depended on the *predictability* of the context as well other factors associated with the stimulus properties (e.g., intelligibility). That a predictive context may lead to either increased or decreased activity as a function of the intelligibility of the stimulus (in temporal and/or parietal areas) (McGettigan et al., 2012; Guediche et al., 2013) suggests that the generation of prediction error signals may be informed by the integration of multiple sources of information, and not solely by the computation derived from a predictive context.

Above, we suggested that adaptive plasticity is guided by supervisory signals derived from discrepancies between expected and actual sensory input. The evidence we reviewed from recent studies in speech perception examining predictive coding in online speech perception is beginning to reveal the cortical networks engaged by tasks that manipulate signal distortions and predictive contexts. To date, the findings related to interactions between predictive contexts and stimulus distortions provide support for a dynamic speech processing framework where predictions can be generated from contextual sources of information and be used to derive prediction error signals. In the predictive coding framework the error signal presumably is used to optimize future predictions and drive learning mechanisms that lead to adaptive plasticity (Clark, 2013). Despite potential similarities between the mechanisms underlying these effects [although see Norris et al. (2003) for a different view], adaptive plasticity differs from the online effects of predictive context on interpreting distorted speech acoustics in that it impacts subsequent perception of speech even once disambiguating contexts are no longer available. While it is possible that predictive coding provides a means of generating prediction error signals that can be used to supervise adaptive plasticity, it is not clear how changes in activity related to predictive coding could give rise to the adaptive plasticity effects evident in the behavioral literatures reviewed above. Although many details about prediction-error-signal driven learning remain to be discovered, it is uncontroversial that the brain integrates incoming sensory information with prior perceptual, motor, and cognitive knowledge to arrive at a unified perceptual experience.

### Neuroimaging evidence for adaptive plasticity in speech perception

In an attempt to dissociate neural changes directly related to adaptive plasticity from modulatory effects of factors such as predictive context and stimulus distortions, we review studies that have specifically investigated changes in neural activity associated with adaptive plasticity (Adank and Devlin, 2010; Eisner et al., 2010; Kilian-Hutten et al., 2011a,b; Erb et al., 2013). Although tasks (word recognition and acoustic phonetic perception) and stimulus manipulations (noise-vocoded, time-compressed, ambiguous) vary across these studies, collectively they implicate the involvement of premotor, temporal, parietal, and frontal areas in adaptive speech perception.

In word recognition studies, evidence for the recruitment of temporal and premotor areas is consistent across studies. Adank and Devlin (2010) examined adaptive plasticity during exposure to time-compressed speech and showed increased activation in bilateral auditory cortex and left ventral premotor cortex associated with adaptation. They concluded that under adverse listening conditions, such as time compression, the dorsal motor stream is recruited to facilitate disambiguation of the speech signal. In a recent word recognition study, Erb et al. (2013) showed that greater changes in activity in precentral gyrus were associated with greater adaptive plasticity after exposure to a noise-vocoded speech distortion. The involvement of the motor system is consistent with prior work suggesting that motor recruitment may facilitate the resolution of perceptually ambiguous speech signals under difficult listening conditions (e.g., Davis and Johnsrude, 2003, 2007; Rauschecker, 2011; Szenkovitz et al., 2012).

The recruitment of other regions may also be important for adaptive plasticity. Eisner et al. (2010) examined adaptation to a speech distortion that simulated cochlear-implant speech input and found that activity in superior temporal cortex and inferior frontal gyrus corresponded with improvements in intelligibility with training. They also found that learning over the course of the experiment corresponded to modulation of activity in a parietal area—specifically, the angular gyrus. The angular gyrus may be ideally suited for guiding the adaptation process, as its functional and structural connectivity with other brain regions suggests that it may provide a point of convergence for motor, sensory, and more abstract linguistic information (Binder et al., 2009; Friederici, 2009; Turken and Dronkers, 2011). Guediche et al. (accepted) also showed differences in frontal and temporal areas before vs. after adaptation to vocoded and spectrally-shifted speech. Taken together, changes in frontal, temporal, and premotor areas have been associated with manipulations of disambiguating contexts context and the severity/intelligibility of the distorted stimuli.

Fewer studies have investigated visually- and lexically-mediated adaptive plasticity of acoustic phonetic perception using neuroimaging. One study examined visually-mediated adaptive plasticity using videos of articulating faces to disambiguate ambiguous acoustic speech stimuli (Kilian-Hutten et al., 2011a). As in the behavioral study by Bertelson et al. (2003), exposure to an ambiguous token paired with a video of a face clearly articulating one of the phonetic alternatives led the ambiguous token to be perceived more often as the alternative consistent with the articulating face in a later acoustic phonetic perception task. Kilian-Hutten et al. (2011a) showed that the perceptual interpretation of the ambiguous sounds could be decoded with multi-voxel pattern analysis in temporal areas (adjacent to and encompassing Heschl's gyrus). This demonstrates a change in the neural pattern of activity consistent with the perceptual change relatively early in auditory cortical networks caused by adaptive plasticity. In order to identify regions involved in learning, Kilian-Hutten et al. (2011b) examined how brain activity during adaptation was related to later perception of the ambiguous stimuli. They found that the visually-mediated adaptive plasticity of acoustic phonetic perception corresponded to changes in activity in a network of areas including frontal, temporal, and parietal areas (Kilian-Hutten et al., 2011b).

To our knowledge, only one neuroimaging study has examined lexically-mediated adaptive plasticity in acoustic phonetic perception (Mesite and Myers, 2012). Similar to the behavioral study by Kraljic and Samuel (2005), two groups of participants were exposed to ambiguous [s]-[∫] tokens in different biasing lexical contexts. They showed between-group changes in subsequent acoustic phonetic perception of the ambiguous tokens presented without lexically-disambiguating contexts. The behavioral changes in acoustic phonetic perception were associated with differences in the activity of right frontal and middle temporal areas. The limited data that exist thus suggest that, similar to the findings from word recognition studies, adaptive plasticity evidenced in acoustic phonetic perception of ambiguous phonetic categories engages a network of frontal, temporal, and parietal areas.

Because of the use of different stimuli, tasks (examining context effects vs. adaptation effects), and analyses (focusing on specific changes and sometimes specific regions) across studies, many questions remain open. Furthermore, even though there is a great deal of evidence supporting the multiple stream view of speech processing, there is still debate regarding the role of specific regions in speech and language processes. Despite these caveats, the current evidence is consistent with a view that frontal (e.g., inferior frontal and middle frontal gyrus) and temporal areas (e.g., superior temporal and middle temporal gyrus) are sensitive to context and stimulus properties. Frontal areas may provide the source of the predictive feedback, potentially involving different frontal areas for different sources of contextual information (Rothermich and Kotz, 2013) and may modulate activity in temporal areas associated with earlier perceptual processes (Gagnepain et al., 2012). Changes in brain activity related to adaptive plasticity may rely more specifically on the recruitment of higher association areas (e.g., parietal cortex) that seem to relate more directly to adaptive plasticity (Obleser et al., 2007; Eisner et al., 2010; Guediche et al., accepted). In all, the literatures investigating the neural basis of predictive coding and adaptive plasticity complement one another and can be leveraged for developing and refining a more detailed model of the dynamic, flexible nature of speech perception.

Despite these advances in our understanding of how specific cortical regions may contribute to a dynamically adaptive speech perception network, presently, there is no formal speech perception model that relates activity in the cortical regions identified via neuroimaging to the computational demands of adaptive plasticity in speech perception. Conversely, the classic computational models of speech perception that have attempted to differentiate how the system may meet the computational demands of adaptive plasticity have not made specific predictions of the underlying neural mechanisms. Next-generation models will need to bridge this divide to explain how adaptive changes in perception are reflected in brain activity and how they take place without undermining the stability of and sensitivity to long-term regularities.

We next examine literatures outside of speech perception for insight into how we may make progress toward meeting these challenges. Inasmuch as it relates to the dual demands of maintaining long-term representations that respect regularities of the environment while flexibly adjusting perception to short-term deviations from these regularities, adaptive plasticity is not unique to speech perception. Preserving the balance between stability and plasticity is important for perceptual, motor and cognitive processing in many domains. Consequently, research outside the domain of speech perception may provide insight regarding the development of a biologically plausible account of adaptive plasticity in speech processing that captures the significant behavioral characteristics we outlined above.

## Insights from neuroscience

Thus far, research on the neural basis of adaptive plasticity in speech perception has been largely focused on cerebral cortical regions. In the section that follows, we argue that the cerebellum plays a role in adaptive plasticity in speech perception. Specifically, we review evidence from sensorimotor learning for cerebellar involvement in perception, predictive coding, and adaptive plasticity. We consider the potential importance of cerebro-cerebellar interactions in generating prediction errors derived from discrepancies between predicted and actual sensory input. Such a mechanism may provide a way to unite the seemingly distinct behavioral speech perception phenomena we reviewed above. Finally, we propose that such a mechanism may be especially relevant since it offers a means to achieve rapid adjustment of perception in response to short-term deviations without undermining the stability of learned long-term regularities.

It may seem surprising to consider the cerebellum as part of a network involved in perceptual plasticity as, historically, the cerebellum has been considered a primarily motor structure. Since many neuroimaging studies of speech perception are focused on changes in perisylvian areas, data collection and/or analyses often fail to consider the cerebellum. However, outside the domain of speech perception, there has been increased interest in the cerebellum's role in non-motoric functions, with some limited but compelling evidence that it is involved in cognitive functions, including language (Fiez et al., 1992; Desmond and Fiez, 1998; Thach, 1998; Strick et al., 2009; although see Glickstein, 2006 for debate). This perspective posits that the cerebellar system plays an important role in supervised learning across many different domains through the manipulation of internal models (Ito, 2008). We next briefly review evidence for cerebellar involvement in sensorimotor adaptation.

### Cerebellar-dependent supervised learning in sensorimotor tasks

In the sensorimotor domain, the underlying mechanisms of adaptation to sensory input distortions have been explored extensively, with multiple lines of evidence underscoring the significance of the cerebellum. A classic behavioral task demonstrating sensorimotor adaptation is visually-guided reaching while wearing prism goggles (e.g., Martin et al., 1996b). When prism goggles that shift the visual field several degrees distort sensory input, motor behavior in a visually guided reaching task is impacted. Initially, reaches are off-target. However, participants rapidly adapt to the distorted sensory input across 10–20 reaches, as evidenced by successful on-target reaching (Martin et al., 1996b). Such sensorimotor adaptation is observed across many stimulus distortions and motor behaviors (Kawato and Wolpert, 1998; Wolpert et al., 1998). Clinical studies examining performance on sensorimotor tasks in patients with cerebellar damage (Martin et al., 1996a; Ackermann et al., 1997), functional neuroimaging studies examining changes in neural activity in short-term adaptation tasks (Clower et al., 1996), and lesion studies with non-human primates (Kagerer et al., 1997; Baizer et al., 1999) all implicate the cerebellum as having an important role in such sensorimotor adaptation.

The role of the cerebellum in sensorimotor adaptation has been attributed largely to supervised learning mechanisms based on internally-generated sensory prediction errors (e.g., Doya, 2000; Shadmehr et al., 2010). Cerebellar-dependent supervised learning within the context of sensorimotor adaptation is thought to rely on the internal generation of sensory prediction error signals derived from discrepancy between the predicted and actual sensory input (Wolpert et al., 2011). The predicted sensory input is the expected outcome of a planned movement (a reach, for example) and can thus be derived from the “internal model” of the input-output relationship of sensory and motor information. With repeated visually-guided reaches while wearing prism goggles, for example, the sensory prediction errors reconfigure the relationship among visual, motor, and proprioceptive information sources to optimize future predictions and minimize error signals, leading to adaptation evidenced by more accurate reaching on subsequent trials (Kawato and Wolpert, 1998; Bedford, 1999; Desmurget and Grafton, 2000; Flanagan et al., 2003; Scott and Wise, 2004; Shadmehr et al., 2010; Clark, 2013).

Such sensorimotor adaptation is also evident in the domain of speech. Adaptation is observed when speakers experience sensory input distortions while talking, such as through real-time manipulation of voice acoustics to alter acoustic feedback from one's own voice or via somatosensory perturbations that alter the feel of speech articulation (e.g., Houde and Jordan, 1998, 2002; Perkell et al., 2007; Villacorta et al., 2007; Shiller et al., 2009; Golfinopoulos et al., 2011; Chang et al., 2013). Speakers quickly adjust their production in a direction that compensates for the sensory input distortion (Houde and Jordan, 1998). In this way, speech production exhibits compensatory motor changes in response to distorted sensory input just as observed for other sensorimotor tasks (Houde and Jordan, 1998, 2002; Jones, 2003). A range of acoustic manipulations has been examined including shifts in fundamental frequency, vowel formant frequency, and the timing of auditory speech feedback (Houde and Jordan, 1998; Jones and Munhall, 2000; Perkell et al., 2007). These shifts can be quite extreme. In one study, participants produced a completely different vowel sound relative to the intended target after they were exposed to vowel formant shifts (Houde and Jordan, 1998).

Neuroanatomical models of speech production have incorporated the idea of internal models that represent the relationship between the sensory input and motor output (Guenther, 1995; Guenther and Ghosh, 2003; Kotz and Schwartze, 2010; Tian and Poeppel, 2010; Price et al., 2011). Guenther (1995); Guenther and Ghosh (2003) developed a neuroanatomically-based computationally model of speech production that incorporates expected relationships between a desired sensory outcome, the motor commands that should produce this outcome, and the actual sensory consequences of the produced speech. The DIVA (Directions Into Velocities of Articulators) model consists of several cerebral cortical areas that interact with the cerebellum, forming a network that guides sensorimotor adaptation in speech production. Through these interactions, internal models can be used to detect and correct errors under sensory input perturbations. Neuroimaging studies of sensorimotor adaptation in speech production have yielded results consistent with predictions from this model. In a study that investigated somatosensory perturbations by using a device to block jaw movement, increases in the BOLD signal were observed across left inferior frontal gyrus, ventral premotor cortices, supramarginal gyri, and the cerebellum, consistent with the model's predictions. These results provided support for the view that cerebro-cerebellar interactions are involved in sensorimotor adaptation in speech (Golfinopoulos et al., 2011). A recent study by Zheng et al. (2013) suggests that multiple interacting functional networks are involved in coding different aspects of the error signals. As reviewed briefly above, although the speech production literature has focused largely on cerebral cortical areas (e.g., Price et al., 2011; but see Guenther and Ghosh, 2003), there is convergent evidence from other literatures that supervised prediction error learning involves cerebro-cerebellar interactions (Doya, 2000; Ito, 2008; Wolpert et al., 2011). In the current speech production models, generation of prediction error signals may relate to those in speech perception either through the sensory expectations that are generated from internal speech processes (e.g., Tian and Poeppel, 2010) or from phonological information (e.g., Price et al., 2011).

There is still debate regarding the role of the motor system in generating predictions during speech perception. Pickering and Garrod (2007) suggested that multiple levels of linguistic information (e.g., semantic, syntactic) engage speech production processes to generate predictions. More recently, Tian and Poeppel (2013) instructed participants to engage in overt speaking, covert/imagined speaking, or imagined hearing and found that there may be differences in how predictions are generated depending on the nature of the speaking tasks participants were engaged in. Tian and Poeppel (2013) suggest that linguistic information retrieved from memory, as well as inner speech processes, can be used to generate predictions and modulate activity in regions associated with perceptual processes. This is consistent with models of visual perception, which also suggest that multiple sources of information can provide feedback to early visual areas (Mumford, 1992; Rao and Ballard, 1999). Thus, cerebellar-dependent supervised learning mechanisms may contribute to adaptive plasticity in speech perception that may operate on prediction error signals derived directly from different sources of linguistic information, indirectly from inner speech motor processes, or both.

Although the focus of research has been, and continues to be, on cerebellar contributions to the adaptive control of movement through sensorimotor adaptation, there is mounting evidence that the cerebellum is also involved in many other perceptual (Ivry, 1996; Petacchi et al., 2005) and cognitive behaviors (Fiez et al., 1992; Desmond and Fiez, 1998; Thach, 1998; Strick et al., 2009). At the outset, we noted that the cerebellum is increasingly recognized to play an important role in supervised learning, across many domains, through the manipulation of internal models (Ito, 2008). In sensorimotor learning, sensory prediction errors realign internal models of sensorimotor relationships. If the role of the cerebellum is more general, it is possible that it is involved in supervised learning that serves to align sensory input with predictions arising from *nonmotor* sources thus extending cerebellar-dependent supervised learning outside sensorimotor domains, (e.g., Doya, 2000; Ito, 2008; Strick et al., 2009).

Indeed, in a nonmotor perceptual task, recent evidence points to cerebellar involvement in perception of spatiotemporal relationships. Roth et al. (2013) recently demonstrated that cerebellar patients are impaired in their ability to adapt to discrepancies in a nonmotor task that relies on spatio-temporal judgments about a visual target. This study provides direct evidence of cerebellar involvement in perceptual adaptation within an entirely nonmotor task that is not dependent on the consequences of one's own motor behavior. There is also evidence that the cerebellum is involved in encoding acoustic sensory prediction error signals in a nonmotor task. Schlerf et al. (2012) showed that activity in the cerebellum is modulated by sensory changes in an acoustically presented stimulus (Schlerf et al., 2012), and different forms of predictive information (Rothermich and Kotz, 2013). In sum, intriguing recent results, even outside the domain of speech perception, suggest the possibility of cerebellar involvement in supervised learning that extends beyond sensorimotor interactions.

In light of known interactions between perception and production, a relationship between the mechanisms that underlie sensorimotor and sensory adaptation seems likely. In fact, even sensorimotor adaptation can evoke “purely” perceptual shifts that are unaccounted for by changes in motor output (e.g., Shiller et al., 2009; Nasir and Ostry, 2009; Mattar et al., 2011). For example, Shiller et al. (2009) demonstrated that after sensorimotor adaptation of speech production induced by altered auditory feedback of a listener's own /∫/ (as in *ship*) productions, subsequent perception of another talker's /s/-/∫/ (as in *sip* to *ship*) sounds was also shifted. Thus, the consequences of sensorimotor adaptation (attributed to cerebellar supervised learning mechanisms) may have a perceptual component that is unrelated to changes in motor output.

The link between sensorimotor adaptation and sensory adaptation, together with recent evidence implicating the cerebellum in purely perceptual adaptation (e.g., Roth et al., 2013) suggest that the supervised learning mechanisms posited for sensorimotor adaptation in speech (Houde and Jordan, 1998; Jones and Munhall, 2000; Guenther and Ghosh, 2003; Shiller et al., 2009) can also provide a framework for understanding adaptive plasticity in speech perception. In speech perception, predictions about sensory input may be derived from multiple sources of information (e.g., lexical, visual) that constrain listeners' interpretation of incoming acoustic signals.

Guediche et al. (accepted) recently examined the potential for cerebellar contributions to adaptive plasticity in speech perception. To this end, they examined neural activity linked to improvements in recognition of acoustically distorted words. Several cerebellar regions showed significantly different activation before, compared to after, adaptation to acoustically distorted words. Activity in one region, right Crus I (previously implicated in language tasks; Stoodley and Schmahmann, 2009; Keren-Happuch et al., 2012) was significantly correlated with behavioral improvement measures of adaptive plasticity during the adaptation phase of the experiment. A seed functional correlation analysis revealed that hemodynamic responses in right Crus I during adaptation significantly covaried with areas in parietal and temporal cortices. This evidence is consistent with prior functional neuroimaging findings implicating these cerebral cortical regions in adaptive plasticity (e.g., Eisner et al., 2010), and extends those prior findings to include the cerebellum as part of a cerebro-cortical functional network that contributes to adaptive changes in speech perception.

In sum, the recent theoretical development and empirical investigation of predictive coding and adaptive plasticity in speech processing, as reviewed above, offers a framework for understanding how prediction errors may be computed, represented, and used to optimize perception. Although prior neuroimaging studies of speech perception adaptation and predictive coding have specifically focused on changes in cerebral cortical areas, the converging lines of evidence described above are consistent with the involvement of cerebellar-supervised learning via cerebro-cerebellar interactions. We are proposing that the cerebellum plays a key role in adaptive plasticity and critically provides a mechanism that can allow for plasticity in the context of a stable perceptual system. In particular, the cerebellum provides an established neural mechanism known to be involved in rapid adaptive plasticity. More research will be needed to examine this issue but this hypothesis provides a working framework for examining the dual roles of stability and plasticity in cognitive systems generally, and in speech perception in particular.

Finally, with regard to maintaining stability it is notable that there is evidence for the possibility that the cerebellum (potentially through interactive loops with cerebral cortex) can maintain multiple adaptive adjustments to internal models (Cunningham and Welch, 1994; Martin et al., 1996b; Imamizu et al., 2003). This provides the means for rapid and short-term adaptive plasticity that can be implemented without catastrophically affecting the stability of long-term regularities. Most germane to adaptive plasticity in speech perception, it presents the opportunity for multiple relationships between acoustic input and linguistic information to be simultaneously represented, such as might be necessary to maintain adaptation to different speakers or different accents. Thus, future neuroimaging efforts should be attentive to including the cerebellum (and potentially other subcortical structures) in the network of regions investigated as contributing to adaptive plasticity in speech perception.

### Conclusions and future directions

Everyday speech communication largely takes place in suboptimal or even adverse listening conditions, at least relative to the pristine listening environments in which most research is conducted. The acoustic speech signals most often conveying meaning to listeners in everyday conversation carry the influence of noisy environments, foreign accented talkers, reduced conversational speech, and dysfluency (see Mattys et al., 2012). We have reviewed several parallel behavioral literatures that demonstrate that the perceptual system makes rapid adaptive adjustments in response to distorted acoustic speech input. We make the case that these largely unconnected behavioral literatures, which have focused on different aspects of speech processing (spoken word recognition and acoustic-phonetic perception) may, in fact, be linked by common factors. We have reviewed computational modeling in the speech perception and neuroscience literatures within and outside the field of speech communication. We have considered how these literatures speak to prospective mechanisms and their ability to unite the behavioral literatures on adaptive plasticity in word recognition and acoustic phonetic perception. In addition, we considered two separate, but complementary, neuroimaging literatures on predictive coding and adaptive plasticity, with the goal of informing the mechanistic basis of adaptive plasticity in speech perception. Both predictive coding and adaptive plasticity models posit mechanisms for encoding error signals when there is a discrepancy between predicted and actual sensory input. Supervised learning mechanisms that rely on prediction error signals for rapid adaptive plasticity have been well-established in the sensorimotor literature, including speech production adaptation tasks, and have been attributed to cerebro-cerebellar interactions. More recently, they have been implicated in nonmotor, perceptual tasks including speech perception. We posit that these findings suggest prediction error-driven learning orchestrated via cerebro-cerebellar interactions may play a role in adaptive plasticity in speech perception.

Based on the synthesis of these literatures, we argued that the generation of predictions, prediction error signals, and supervised learning may be significant in driving adaptive plasticity. In particular, we highlighted the potential for a cerebellar-dependent supervised learning mechanism to play a role in adaptive plasticity in speech perception and described preliminary evidence that supports this possibility. This perspective suggests some directions for future research that will better develop neurobiological models of speech communication that capture the dynamic, online flexibility of the system.

Although a great deal of evidence points to the importance of subcortical-cortical interactions in adaptive plasticity in other domains, the mainstream literature on speech perception has yet to make significant contact with the literature on subcortical contributions to adaptive plasticity. Neuroscience research relevant to adaptive plasticity in speech perception and, indeed to speech perception more generally, has tended to be be focused on the cerebrum. Although we know less about contributions of subcortical structures in speech perception, there have been a number of studies that have highlighted roles for the cerebellum, thalamus, caudate, and the brainstem that may be defined by specific functions, or interactions with specific regions in cerebral cortex (Ravizza, 2003; Tricomi et al., 2006; Song et al., 2008, 2011, 2012; Stoodley and Schmahmann, 2009; Anderson and Kraus, 2010; Stoodley et al., 2012; Erb et al., 2013).

In the broader neuroscience literature, developing perspectives have suggested that different types of learning mechanisms may be subserved by different neural systems. At least three types of potentially distinct and interacting learning circuits have been proposed for unsupervised, reinforcement, and supervised learning (see Doya, 2000; Hoshi et al., 2005; Bostan et al., 2010; Wolpert et al., 2011). Doya (2000) suggested that unsupervised learning algorithms depend mostly on long-term changes in cerebral cortex that can be incorporated over longer timecourses (Doya, 2000). Reinforcement learning, on the other hand, relies on information to predict reward outcomes. In speech perception, reinforcement learning has been examined in the context of non-native category learning. In a functional neuroimaging study, Tricomi et al. (2006) examined learning with performance feedback and found that basal ganglia activity was modulated by the presence of feedback during a non-native phonetic category perception task just as they are in other reinforcement learning tasks (e.g., Delgado et al., 2000). Whereas reinforcement learning may optimize subsequent reward prediction error and engage the basal ganglia, supervised learning may optimize sensory prediction error signals by engaging the cerebellum.

In speech perception, both unsupervised and supervised learning mechanisms have been used to account for adaptive plasticity (Norris et al., 2003; Mirman et al., 2006). Outside the domain of speech perception, unsupervised learning mechanisms are generally used to model learning that arises over longer time courses (McClelland et al., 1995; O'Reilly, 2001; Grossberg, 2013) than the learning that characterizes adaptive plasticity. Supervised learning in models of speech perception have not accounted for many known behavioral and biological constraints, However, outside the domain of speech perception, recent models have explored a number of alternatives for achieving neurobiologically plausible supervised learning algorithms (e.g., Yu et al., 2008; Chinta and Tweed, 2012).

In speech, there is behavioral evidence that listeners can achieve greater levels of adaptation that go beyond those reached with rapid adaptation training paradigms, if they are exposed to multiple sessions with consolidation (Banai and Lavner, 2012). Improvements in word recognition for distorted acoustic input degrade over the course of a day-long retention interval, but are fully restored with sleep; sleep thus appears to stabilize what is learned in adaptation to distorted speech (Fenn et al., 2003), with word recognition improvements lasting as long as 6 months (Schwab et al., 1985). Thus, a fully mechanistic account of speech processing will require an understanding of how and to what extent different learning mechanisms interact with one another to influence speech processing. Some computational accounts of perception have begun to incorporate different types of learning algorithms within single systems (Hinton and Plaut, 1987; O'Reilly, 2001; Kleinschmidt and Jaeger, 2011; Grossberg, 2013). One challenge for models of speech processing is to account for the equilibrium that must be maintained between mechanisms involved in preserving stability while supporting plasticity.

In light of the parallels we have drawn between adaptive plasticity in speech perception and sensorimotor adaptation, it is interesting to note that research has demonstrated retention of sensorimotor adaptation effects over more than a year (Yamamoto et al., 2006) suggesting that cerebellar-dependent supervised learning can evoke changes in internal models that are maintained across long time periods. Yamamoto et al. speculate that the extent to which sensorimotor adaptation is retained depends on an interaction between the number of training trials and the magnitude of the distortion, with more subtle distortions leading to longer-lasting adaptation perhaps because they evoke smaller errors and avoid engaging explicit compensation mechanisms (Redding and Wallace, 1996). These issues have not been investigated in the adaptive plasticity of speech perception, but have important implications for long-lasting adaptation in speech perception. Understanding the details of the interplay between the different types of learning mechanisms will be crucial for understanding how the system maintains balance between stability and plasticity in speech perception.

Beyond delineating the learning mechanisms available to guide adaptive plasticity in speech perception, there also are many open questions regarding the nature of putative prediction errors and how predictions may be derived from various information sources. The field has focused much attention on the role of lexical information in driving adaptive plasticity. Other sources of information, such as co-speech gestures from arm and hand movements associated with speech communication (Skipper et al., 2009), semantic or sentence context (e.g., Borsky et al., 1998; Zekveld et al., 2012), knowledge about the speaker (Samuel and Kraljic, 2009), or previously learned voice-face associations (von Kriegstein et al., 2008) may provide a basis for disambiguating distorted acoustic input via prediction errors and, potentially, may drive adaptive plasticity. Indeed, in more natural communication, many different information sources converge to constrain predictions and disambiguate acoustic speech input. The emerging framework we have begun to sketch unites the means by which these very different information sources drive adaptive plasticity in speech perception. These other sources of information provide a constraint on the predictions the system makes about the intended message and, in turn, affect the sensory prediction that is made and the prediction error that results. Moreover, since both internally-generated and external sensory input inform predictions, it becomes easier to reconcile seemingly distinct influences of acoustic sensory distortions and higher-level influences such as expectations about speaker- or context-specific factors that influence speech, (Kraljic et al., 2008; Kraljic and Samuel, 2011).

In conclusion, evidence for a flexible speech perception system that rapidly adapts to accommodate systematic distortions in acoustic speech input is abundant. A review of behavioral, computational, and neuroscience research related to rapid adaptive mechanisms suggests that it may be informative to consider phenomena in literatures outside of speech communication to identify common and unifying principles of how the brain balances stability and plasticity. Here, we examined cerebellar-dependent supervised learning that relies on sensory prediction error signals as a potential mechanism for supervising adaptive changes in speech perception. The predictions used to derive the error signals may be generated from multiple interacting sources of external sensory and internally-generated information. By incorporating cerebral-subcortical interactions established in other literatures into neuroanatomical theories of speech perception, the mechanisms that contribute to stability and plasticity may be better understood.

### Conflict of interest statement

The authors declare that the research was conducted in the absence of any commercial or financial relationships that could be construed as a potential conflict of interest.

## References

[B1] Ackermann H. Graber S. Hertrich I. Daum I. (1997). Categorical speech perception in cerebellar disorders. Brain Lang. 60, 323–331 10.1006/brln.1997.1826 9344481

[B2] Adank P. Devlin J. (2010). On-line plasticity in spoken sentence comprehension: adapting to time-compressed speech. Neuroimage 49, 1124–1132 10.1016/j.neuroimage.2009.07.032 19632341 PMC2775905

[B3] Adank P. Janse E. (2009). Perceptual learning of time-compressed and natural fast speech. J. Acoust. Soc. Am. 126, 2649–2659 10.1121/1.3216914 19894842

[B4] Altmann G. T. M. Young D. (1993). Factors affecting adaptation to time-compressed speech, in Third European Conference on Speech Comunication, (Berlin).

[B5] Anderson S. Kraus N. (2010). Sensory-cognitive interaction in the neural encoding of speech in noise. J. Am. Acad. Audiol. 21, 575–585 10.3766/jaaa.21.9.3 21241645 PMC3075209

[B6] Baizer J. S. Kralj-Hans I. Glickstein M. (1999). Cerebellar lesions and prism adaptation in macaque monkeys. J. Neurophysiol. 81, 1960–1965 10200230 10.1152/jn.1999.81.4.1960

[B7] Banai K. Lavner Y. (2012). Perceptual learning of time-compressed speech: more than rapid adaptation. PLoS ONE 7:e47099 10.1371/journal.pone.0047099 23056592 PMC3467231

[B8] Bastos A. Usrey W. Adams R. Mangun G. Fries P. Friston K. (2012). Canonical microcircuits for predictive coding. Neuron 76, 695–711 10.1016/j.neuron.2012.10.038 23177956 PMC3777738

[B9] Bedford F. L. (1999). Keeping perception accurate. Trends Cogn. Sci. (Regul. Ed). 3, 4–11 10.1016/S1364-6613(98)01266-2 10234221

[B10] Bertelson P. Vroomen J. Gelder B. D. (2003). Visual recalibration of auditory speech identification: a McGurk aftereffect. Psychol. Sci. 14, 592–597 10.1046/j.0956-7976.2003.psci_1470.x 14629691

[B11] Bertelson P. Vroomenti J. Gelderti B. (1997). Auditory-visual interaction in voice localization and in bimodal speech recognition: the effects of desynchronization, in AVSP-1997 (Rhodes), 97–100

[B12] Binder J. R. Desai R. H. Graves W. W. Conant L. L. (2009). Where is the semantic system? a critical review and meta-analysis of 120 functional neuroimaging studies. Cereb. Cortex 19, 2767–2796 10.1093/cercor/bhp055 19329570 PMC2774390

[B13] Binder J. R. Liebenthal E. Possing E. T. Medler D. A. Ward B. D. (2004). Neural correlates of sensory and decision processes in auditory object identification. Nat. Neurosci. 7, 295–301 10.1038/nn1198 14966525

[B14] Blumstein S. Myers E. Rissman J. (2005). The perception of voice onset time: an fMRI investigation of phonetic category structure. J. Cogn. Neurosci. 17, 1353–1366 10.1162/0898929054985473 16197689

[B15] Borsky S. Tuller B. Shapiro L. (1998). “How to milk a coat:” the effets of semantic and acoustic information on phoneme categorization. J. Acoust. Soc. Am. 103, 2670–2676 10.1121/1.422787 9604360

[B16] Bostan A. C. Dum R. P. Strick P. L. (2010). The basal ganglia communicate with the cerebellum. Proc. Natl. Acad. Sci. U.S.A. 107, 8452–8456 10.1073/pnas.1000496107 20404184 PMC2889518

[B17] Bradlow A. Bent T. (2008). Perceptual adaptation to non-native speech. Cognition 106, 707–729 10.1016/j.cognition.2007.04.005 17532315 PMC2213510

[B18] Cainer K. James C. Rajan R. (2008). Learning speech-in-noise discrimination in adult humans. Hear. Res. 238, 155–164 10.1016/j.heares.2007.10.001 18024026

[B19] Caplan D. (1999). Activating brain systems for syntax and semantics. Neuron 24, 292–293 10.1016/S0896-6273(00)80843-0 10571223

[B20] Caplan D. (2006). Why is Broca's area involved in syntax? Cortex 24, 42</page>, <page>469–471 10.1016/S0010-9452(08)70379-4 16881251

[B21] Chang E. Niziolek C. Knight R. Nagarajan S. Houde J. (2013). Human cortical sensorimotor network underlying feedback control of vocal pitch. Proc. Natl. Acad. Sci. U.S.A. 110, 2653–2658 10.1073/pnas.1216827110 23345447 PMC3574939

[B22] Chinta L. V. Tweed D. B. (2012). Adaptive optimal control without weight transport. Neural Comput. 24, 1487–1518 10.1162/NECO_a_00277 22364503

[B23] Clark A. (2013). Whatever next? Predictive brains, situated agents and the future of cognitive science. Behav. Brain Sci. 36, 131–204 10.1017/S0140525X12000477 23663408

[B24] Clarke C. Garrett M. (2004). Rapid adaptation to foreign-accented English. J. Acoust. Soc. Am. 116, 3647–3658 10.1121/1.1815131 15658715

[B25] Clarke-Davidson C. Luce P. Sawusch J. (2008). Does perceptual learning in speech reflect changes in phonetic category representation or decision bias. Percept. Psychophys. 70, 604–618 10.3758/PP.70.4.604 18556922

[B26] Clayards M. Tanenhaus M. K. Aslin R. N. Jacobs R. A. (2008). Perception of speech reflects optimal use of probabilistic speech cues. Cognition 108, 804–809 10.1016/j.cognition.2008.04.004 18582855 PMC2582186

[B27] Clos M. Langner R. Meyer M. Oechslin M. S. Zilles K. Eickhoff S. B. (2012). Effects of prior information decoding degraded speech: an fMRI study. Hum. Brain Mapp. 35, 61–74 10.1002/hbm.22151 22936472 PMC6868994

[B28] Clower D. M. Hoffman J. M. Votaw J. R. Faber T. L. Woods R. P. Alexander G. E. (1996). Role of posterior parietal cortex in the recalibration of visually guided reaching. Nature 383, 618–621 10.1038/383618a0 8857536

[B29] Crick F. (1989). The recent excitement about neural networks. Nature 337, 129–132 10.1038/337129a0 2911347

[B30] Cunningham H. A. Welch R. B. (1994). Multiple concurrent visual-motor mappings:implications for models of adaptation. J. Exp. Psychol. Hum. Percept. Perform. 20, 987–999 10.1037/0096-1523.20.5.987 7964533

[B31] Cutler A. (2008). The abstract representations in speech processing. Q. J. Exp. Psychol. 61, 1601–1619 10.1080/13803390802218542 18671170

[B32] Davis M. Johnsrude I. Hervais-Aldelman A. Taylor K. McGettigan C. (2005). Lexical information drives perceptual learning of distorted speech: evidence from the comprehension of noise-vocoded sentences. J. Exp. Psychol. Gen. 134, 222–241 10.1037/0096-3445.134.2.222 15869347

[B33] Davis M. H. Ford M. A. Kherif F. Johnsrude I. S. (2011). Does semantic context benefit speech understanding through “top-down” processes? Evidence from time-resolved sparse fMRI. J. Cogn. Neurosci. 23, 3914–3932 10.1162/jocn_a_00084 21745006

[B34] Davis M. H. Johnsrude I. S. (2003). Hierarchical processing in spoken language comprehension. J. Neurosci. 23, 3423 10.1093/cercor/bhi009 12716950 PMC6742313

[B35] Davis M. H. Johnsrude I. S. (2007). Hearing speech sounds: top-down influences on the interface between audition and speech perception. Hear. Res. 229, 132–147 10.1016/j.heares.2007.01.014 17317056

[B36] Delgado M. Nystrom L. Fissell C. Noll D. Fiez J. (2000). Tracking the hemodynamic responses to reward and punishment. J. Neurophysiol. 84, 3072–3077 10.1196/annals.1390.002 11110834

[B37] Desmond J. E. Fiez J. A. (1998). Neuroimaging studies of the cerebellum: language, learning and memory. Trends Cogn. Sci. (Regul. Ed). 2, 355–358 10.1016/S1364-6613(98)01211-X 21227232

[B37b] Desmurget M. Grafton S. (2000). Forward modeling allows feedback control for fast reaching movements. Trends Cogn. Sci. 4, 423–431 10.1016/S1364-6613(00)01537-0 11058820

[B37a] DeWitt I. Rauschecker J. P. (2012). Phoneme and word recognition in the auditory ventral stream. Proc. Natl. Acad. Sci. U.S.A. 109, E505–E514 10.1073/pnas.1113427109 22308358 PMC3286918

[B38] Doya K. (2000). Complementary roles of basal ganglia and cerebellum in learning and motor control. Curr. Opin. Neurobiol. 10, 732–739 10.1016/S0959-4388(00)00153-7 11240282

[B39] Eisner F. McGettigan C. Faulkner A. Rosen S. Scott S. (2010). Inferior frontal gyrus activation predicts individual differences in perceptual learning of cochlear-implant simulations. J. Neurosci. 30, 7179–7186 10.1523/JNEUROSCI.4040-09.2010 20505085 PMC2883443

[B40] Erb J. Henry M. Eisner F. Obleser J. (2013). The brain dynamics of rapid perceptual adaptation to adverse listening conditions. J. Neurosci. 33, 10688–10697 10.1523/JNEUROSCI.4596-12.2013 23804092 PMC6618499

[B41] Fedorenko E. Duncan J. Kanwisher N. (2012). Lexical and syntactic representations in the brain: an fMRI investigation with multi-voxel pattern analyses. Neuropsychologia 50, 499–513 10.1016/j.neuropsychologia.2011.09.014 21945850 PMC3292791

[B42] Fenn K. Nusbaum H. Margoliash D. (2003). Consolidation during sleep of perceptual learning of spoken language. Nature 425, 614–616 10.1038/nature01951 14534586

[B43] Fiez J. A. Petersen S. E. Cheney M. K. Raichle M. E. (1992). Impaired non-motor learning and error detection associated with cerebellar damage. Brain 115(Pt.1), 155–178 10.1093/brain/115.1.155 1559151

[B43a] Flanagan J. Vetter P. Johansson R. Wolpert D. (2003). Prediction precedes control in motor learning. Curr. Biol. 13, 146–150 10.1016/S0960-9822(03)00007-1 12546789

[B45] Francis A. Baldwin K. Nusbaum H. (2000). Effects of training on attention to acoustic cues. Percept. Psychophys. 62, 1668–1680 10.3758/BF03212164 11140187

[B46] Francis A. Nusbaum H. Fenn K. (2007). Effects of training on the acoustic phonetic representation of synthetic speech. J. Speech Lang. Hear. Res. 50, 1445–1465 10.1044/1092-4388(2007/100) 18055767

[B47] Friederici A. D. (2009). Pathways to language: fiber tracts in the human brain. Trends Cogn. Sci. (Regul. Ed). 13, 175–181 10.1016/j.tics.2009.01.001 19223226

[B48] Friston K. Kiebel S. (2009). Predictive coding under the free-energy principle. Philos. Trans. R. Soc. B Biol. Sci. 364, 1211–1221 10.1098/rstb.2008.0300 19528002 PMC2666703

[B49] Fu Q. Galvin J. R. (2003). The effects of short-term training for spectrally mismatched noise-band speech. J. Acoust. Soc. Am. 113, 1065–1072 10.1121/1.1537708 12597199

[B50] Gagnepain P. Henson R. N. Davis M. H. (2012). Temporal predictive codes for spoken words in auditory cortex. Curr. Biol. 22, 615–621 10.1016/j.cub.2012.02.015 22425155 PMC3405519

[B51] Ganong W. R. (1980). Phonetic categorization in auditory word perception. J. Exp. Psychol. Hum. Percept. Perform. 6, 110–125 10.1037//0096-1523.6.1.110 6444985

[B52] Gaskell M. G. Marslen-Wilson W. D. (1997). Integrating form and meaning: a distributed model of speech perception. Lang. Cogn. Process. 12, 613–656 10.1080/016909697386646

[B53] Glickstein M. (2006). Thinking about the cerebellum. Brain 129, 288–290 10.1093/brain/awh728 16434421

[B54] Golfinopoulos E. Tourville J. Bohland J. Ghosh S. Nieto-Castanon A. Guenther F. (2011). fMRI investigation of unexpected somatosensory feedback perturbation during speech. Neuroimage 55, 1324–1338 10.1016/j.neuroimage.2010.12.065 21195191 PMC3065208

[B55] Gow J. D. Segawa J. Ahlfors S. Lin F. (2008). Lexical influences on speech perception: a Granger causality analysis of MEG and EEG source estimates. Neuroimage 43, 614–623 10.1016/j.neuroimage.2008.07.027 18703146 PMC2585985

[B56] Greenspan S. Nusbaum H. Pisoni D. (1988). Perceptual learning of synthetic speech produced by rule. J. Exp. Psychol. Learn. Mem. Cogn. 14, 421–433 10.1037//0278-7393.14.3.421 2969941 PMC3495315

[B57] Grossberg S. (2013). Adaptive resonance theory: how a brain learns to consciously attend, learn, and recognize a changing world. Neural Netw. 37, 1–47 10.1016/j.neunet.2012.09.017 23149242

[B58] Guediche S. Fiez J. A. Holt L. L. (2009). Perceptual learning of distorted speech with and without feedback, in Poster Presented at the 31^st^ Annual Conference of the Cognitive Science Society. (Amsterdam).

[B59] Guediche S. Holt L. L. Laurent P. Lim S. Fiez J. A. (accepted). Evidence for cerebellar contributions to adaptive plasticity in speech perception. Cereb. Cortex. 10.1093/cercor/bht428 PMC4481605 24451660

[B60] Guediche S. Salvata C. Blumstein S. E. (2013). Temporal cortex reflects effects of sentence context on phonetic processing. J. Cogn. Neurosci. 25, 706–718 10.1162/jocn_a_00351 23281778 PMC3612392

[B61] Guenther F. (1995). Speech sound acquisition, coarticulation, and rate effects in a neural network model of speech production. Psychol. Rev. 102, 594–621 10.1037/0033-295X.102.3.594 7624456

[B62] Guenther F. H. Ghosh S. S. (2003). A model of cortical and cerebellar function in speech, in Proceedings of the XVth International Congress of Phonetic Sciences (Barcelona).

[B62a] Hervais-Adelman A. Davis M. H. Johnsrude I. S. Carlyon R. P. (2008). Perceptual learning of noise vocoded words: effects of feedback and lexicality. J. Exp. Psychol. Hum. Percept. Perform. 34, 460–474 10.1037/0096-1523.34.2.460 18377182

[B63] Hervais-Adelman A. G. Davis M. H. Johnsrude I. S. Taylor K. J. Carlyon R. P. (2011). Generalization of perceptual learning of vocoded speech. J. Exp. Psychol. Hum. Percept. Perform. 37, 283–295 10.1037/a0020772 21077718

[B64] Hesselmann G. Sadaghiani S. Friston K. J. Kleinschmidt A. (2010). Predictive coding or evidence accumulation? False inference and neuronal fluctuations. PLoS ONE 5:e9926 10.1371/journal.pone.0009926 20369004 PMC2848028

[B65] Hickok G. Poeppel D. (2004). Dorsal and ventral streams: a framework for understanding aspects of the functional anatomy of language. Cognition 92, 67–99 10.1016/j.cognition.2003.10.011 15037127

[B66] Hickok G. Poeppel D. (2007). The cortical organization of speech processing. Nat. Rev. Neurosci. 8, 393–402 10.1038/nrn2113 17431404

[B67] Hinton G. Plaut D. (1987). Using fast weights to deblur old memories, in Proceedings of the Ninth Annual Conference of the Cognitive Science Society, (Hillsdale, NJ: Earlbaum).

[B68] Holt L. (2005). Temporally nonadjacent nonlinguistic sounds affect speech categorization. Psychol. Sci. 16, 305–312 10.1111/j.0956-7976.2005.01532.x 15828978

[B70] Hoshi E. Tremblay L. Feger J. Carras P. Strick P. (2005). The cerebellum communicates with the basal ganglia. Nat. Neurosci. 8, 1491–1493 10.1038/nn1544 16205719

[B71] Houde J. F. Jordan M. I. (1998). Sensorimotor adaptation in speech production. Science 279, 1213–1216 10.1126/science.279.5354.1213 9469813

[B72] Houde J. F. Jordan M. I. (2002). Sensorimotor adaptation of speech: compensation and adaptation. J. Speech Lang. Hear. Res. 45, 295–310 10.1044/1092-4388(2002/023) 12003512

[B73] Idemaru K. Holt L. L. (2011). Word recognition reflects dimension-based statistical learning. J. Exp. Psychol. Hum. Percept. Perform. 37, 1939–1956 10.1037/a0025641 22004192 PMC3285244

[B74] Imamizu H. Kuroda T. Miyauchi S. Yoshioka T. Kawato M. (2003). Modular organization of internal models of tools in the human cerebellum. Proc. Natl. Acad. Sci. U.S.A. 100, 5461–6566 10.1073/pnas.0835746100 12704240 PMC154367

[B75] Ito M. (2008). Control of mental activities by internal models in the cerebellum. Nat. Rev. Neurosci. 9, 304–313 10.1038/nrn2332 18319727

[B76] Ivry R. B. (1996). The representation of temporal information in perception and motor control. Curr. Opin. Neurobiol. 6, 851–857 10.1016/S0959-4388(96)80037-7 9000026

[B76b] Jones J. A. (2003). Learning to produce speech with an altered vocal tract: the role of auditory feedback. J. Acoust. Soc. Am. 113, 532–543 10.1121/1.1529670 12558289

[B76a] Jones J. A. Munhall K. G. (2000). Perceptual calibration of *F*0 production: evidence from feedback perturbation. J. Acoust. Soc. Am. 108, 1246–1251 10.1121/1.1288414 11008824

[B77] Kagerer F. Contreras-Vidal J. Stelmach G. (1997). Adaptation to gradual as compared with sudden visuo-motor distortions. Exp. Brain Res. 115, 557–561 10.1007/PL00005727 9262212

[B78] Kawato M. Wolpert D. (1998). Internal models for motor control. Novartis Found. Symp. 218, 291–304 9949827 10.1002/9780470515563.ch16

[B79] Keren-Happuch E. Chen S. H. Desmond J. E. (2012). A meta-analysis of cerebellar contributions to higher cognition from PET and fMRI studies. Hum. Brain Mapp. [Epub ahead of print]. 10.1002/hbm.22194 23125108 PMC3866223

[B80] Kilian-Hutten N. Valente G. Vroomen J. Formisano E. (2011a). Auditory cortex encodes the perceptual interpretation of ambiguous sound. J. Neurosci. 31, 1715–1720 10.1523/JNEUROSCI.4572-10.2011 21289180 PMC6623724

[B81] Kilian-Hutten N. Vroomen J. Formisano E. (2011b). Brain activation during audiovisual exposure anticipates future perception of ambiguous speech. Neuroimage 57, 1601–1607 10.1016/j.neuroimage.2011.05.043 21664279

[B82] Kleinschmidt D. Jaeger T. F. (2011). A Bayesian belief updating model of phonetic recalibration and selective adaptation, in Proceedings of the 2nd Workshop on Cognitive Modeling and Computational Linguistics, (Portland, OR: Association for Computational Linguistics), 10–19

[B83] Kotz S. A. Schwartze M. (2010). Cortical speech processing unplugged: a timely subcortico-cortical framework. Trends Cogn. Sci. 14, 392–399 10.1016/j.tics.2010.06.005 20655802

[B84] Kraljic T. Samuel A. Brennan S. (2008). First impressions and last resorts: how listeners adjust to speaker variability. Psychol. Sci. 19, 332–338 10.1111/j.1467-9280.2008.02090.x 18399885

[B85a] Kraljic T. Samuel A. G. (2005). Perceptual learning for speech: is there a return to normal? Cognit. Psychol. 51, 141–178 10.1016/j.cogpsych.2005.05.001 16095588

[B85] Kraljic T. Samuel A. G. (2011). Perceptual learning evidence for contextually-specific representations. Cognition 121, 459–465 10.1016/j.cognition.2011.08.015 21939965 PMC3214006

[B86] Ladefoged P. Broadbent D. E. (1957). Information conveyed by vowels. J. Acoust. Soc. Am. 29, 113–127 10.1121/1.1908694 2525139

[B87] Leff A. Schofield T. Stephan K. Crinion J. Friston K. Price C. (2008). The cortical dynamics of intelligible speech. J. Neurosci. 28, 13209–13215 10.1523/JNEUROSCI.2903-08.2008 19052212 PMC2613508

[B88] Li T. Fu Q. (2006). Perceptual adaptation to spectrally shifted vowels: training with nonlexical labels. J. Assoc. Res. Otolaryngol. 8, 32–41 10.1007/s10162-006-0059-2 17131213 PMC2538416

[B89] Li T. Fu Q. (2010). Effects of spectral shifting on speech perception in noise. Hear. Res. 270, 81–88 10.1016/j.heares.2010.09.005 20868733 PMC3001342

[B90] Li T. Galvin J. R. Fu Q. (2009). Interactions between unsupervised learning and the degree of spectral mismatch on short-term perceptual adaptation to spectrally shifted speech. Ear Hear. 30, 238–249 10.1097/AUD.0b013e31819769ac 19194293 PMC2889179

[B91] Liss J. Spitzer S. Caviness J. Adler C. (2002). The effects of familiarization on intelligibility and lexical segmentation in hypokinetic and ataxic dysarthria. J. Acoust. Soc. Am. 112, 3022–3030 10.1121/1.1515793 12509024 PMC4063207

[B92] Lotto A. Kluender K. (1998). General contrast effects in speech perception: effect of preceding liquid on stop consonant identification. Percept. Psychophys. 60, 602–619 10.3758/BF03206049 9628993

[B93] MacDonald J. McGurk H. (1978). Visual influences on speech perception processes. Percept. Psychohys. 24, 253–257 10.3758/BF03206096 704285

[B94] Martin T. A. Keating J. G. Goodkin H. P. Bastian A. J. Thach W. T. (1996a). Throwing while looking through prisms. I. Focal olivocerebellar lesions impair adaptation. Brain 119, 1183–1198 10.1093/brain/119.4.1183 8813282

[B95] Martin T. A. Keating J. G. Goodkin H. P. Bastian A. J. Thach W. T. (1996b). Throwing while looking through prisms. II. Specificity and storage of multiple gaze-throw calibrations. Brain 119, 1199–1211 10.1093/brain/119.4.1199 8813283

[B96] Mattys S. L. Davis M. H. Bradlow A. R. Scott S. K. (2012). Speech recognition in adverse conditions: a review. Lang. Cogn. Process. 277, 953–978 10.1080/01690965.2012.705006

[B96a] Mattar A. Nasir S. Darainy M. Ostry D. (2011). Sensory change following motor learning. Prog. Brain Res. 191, 31–44 10.1016/B978-0-444-53752-2.00015-1 21741542

[B96b] Maye J. Aslin R. Tanenhaus M. (2008). The weckud wetch of the wast: lexical adaptation to a novel accent. Cogn. Sci. 5, 543–562 10.1080/03640210802035357 21635345

[B96c] Mumford D. (1992). On the computational architecture of the neocortex. Biol. Cybern. 66, 241–251 10.1007/BF00198477 1540675

[B97] McClelland J. Elman J. (1986). The TRACE model of speech perception. Cogn. Psychol. 18, 1–86 10.1016/0010-0285(86)90015-0 3753912

[B98] McClelland J. McNaughton B. O'Reilly R. (1995). Why there are complementary learning systems in the hippocampus and neocortex: insights from the successes and failures of connectionist models of learning and memory. Psychol. Rev. 102, 419–457 10.1037//0033-295X.102.3.419 7624455

[B99] McClelland J. L. Mirman D. Holt L. L. (2006). Are there interactive processes in speech perception? Trends Cogn. Sci. (Regul. Ed) 10, 363–369 10.1016/j.tics.2006.06.007 16843037 PMC3523348

[B100] McGettigan C. Faulkner A. Altarelli I. Obleser J. Baverstock H. Scott S. (2012). Speech comprehension aided by multiple modalities: behavioural and neural interactions. Neuropsychologia 50, 762–776 10.1016/j.neuropsychologia.2012.01.010 22266262 PMC4050300

[B101] McGurk H. Macdonald J. (1976). Hearing lips and seeing voices. Nature 264, 746–748 1012311 10.1038/264746a0

[B102] McQueen J. M. Tyler M. Cutler A. (2012). Lexical retuning of children's speech perception: evidence for knowledge about words' component sounds. Learn. Dev. 8, 317–339 10.1080/15475441.2011.641887

[B103] Mehler J. Sebastian N. Altmann G. Dupoux E. Christophe A. Pallier C. (1993). Understanding compressed sentences: the role of rhythm and meaning. Ann. N.Y. Acad. Sci. 682, 272–282 10.1111/j.1749-6632.1993.tb22975.x 8323119

[B104] Mesite L. Myers E. (2012). An fMRI study of lexically-induced perceptual learning inspeech, in Poster Presented at the 3rd Annual University of Connecticut Language Fest (Storrs, CT).

[B105] Mirman D. McClelland J. Holt L. (2006). An interactive Hebbian account of lexically guided tuning of speech perception. Psychon. Bull. Rev. 13, 958–965 10.3758/BF03213909 17484419 PMC2291357

[B106] Myers E. Blumstein S. (2008). The neural bases of the lexical effect: an fMRI investigation. Cereb. Cortex 18, 278–288 10.1093/cercor/bhm053 17504782

[B106a] Nasir S. M. Ostry D. J. (2009). Auditory plasticity and speech motor learning. PNAS 106, 20470–20475 19884506 10.1073/pnas.0907032106 PMC2771744

[B107] Norris D. (1994). Shortlist: a connectionist model of continuous speech recognition. Cognition 52, 189–234 10.1016/0010-0277(94)90043-418426294

[B108] Norris D. McQueen J. Cutler A. (2000). Merging information in speech recognition: feedback is never necessary. Behav. Brain Sci. 23, 299–325 10.1017/S0140525X00003241 11301575

[B109] Norris D. McQueen J. M. Cutler A. (2003). Perceptual learning in speech. Cogn. Psychol. 47, 204–238 10.1016/S0010-0285(03)00006-9 12948518

[B110] Obleser J. Kotz S. A. (2010). Expectancy constraints in degraded speech modulate the language comprehension network. Cereb. Cortex 20, 633–640 10.1093/cercor/bhp128 19561061

[B111] Obleser J. Kotz S. A. (2011). Multiple brain signatures of integration in the comprehension of degraded speech. Neuroimage 55, 713–723 10.1016/j.neuroimage.2010.12.020 21172443

[B112] Obleser J. Wise R. Dresner M. Scott S. (2007). Functional integration across brain regions improves speech perception under adverse listening conditions. J. Neurosci. 27, 2283–2289 10.1523/JNEUROSCI.4663-06.2007 17329425 PMC6673469

[B113] O'Reilly R. (2001). Generalization in interactive networks: the benefits of inhibitory competition and Hebbian learning. Neural Comput. 13, 1199–1241 10.1162/08997660152002834 11387044

[B114] Pallier C. Sebastian-Galles N. Dupoux E. Christophe A. Mehler J. (1998). Perceptual adjustment to time-compressed speech: a cross-linguistic study. Mem. Cogn. 26, 844–851 10.3758/BF03211403 9701975

[B115] Panichello M. F. Cheung O. S. Bar M. (2012). Predictive feedback and conscious visual experience. Front. Psychol. 3:620 10.3389/fpsyg.2012.00620 23346068 PMC3549576

[B116] Peelle J. Wingfield A. (2005). Dissociations in perceptual learning revealed by adult age differences in adaptation to time-compressed speech. J. Exp. Psychol. Hum. Percept. Perform. 31, 1315–1330 10.1037/0096-1523.31.6.1315 16366792

[B117] Peelle J. E. Johnsrude I. S. Davis M. H. (2010). Hierarchical processing for speech in human auditory cortex and beyond. Front. Hum. Neurosci. 4:51 10.3389/fnhum.2010.00051 20661456 PMC2907234

[B118] Perkell J. Lane H. Denny M. Matthies M. Tiede M. Zandipour M. (2007). Time course of speech changes in response to unanticipated short-term changes in hearing state. J. Acoust. Soc. Am. 121, 2296–2311 10.1121/1.2642349 17471743

[B119] Petacchi A. Laird A. Fox P. Bower J. (2005). Cerebellum and auditory function: an ALE meta-analysis of functional neuroimaging studies. Hum. Brain Mapp. 25, 118–128 10.1002/hbm.20137 15846816 PMC6871682

[B120] Peterson G. E. Barney H. L. (1952). Control methods used in a study of the vowels. J. Acoust. Soc. Am. 24, 175–184 10.1121/1.1906875

[B120a] Pickering M. Garrod S. (2007). Do people use language production to make predictions during comprehension? Trends Cogn. Sci. 11, 105–110 10.1016/j.tics.2006.12.002 17254833

[B121] Poldrack R. Temple E. Protopapas A. Nagarajan S. Tallal P. Merzenich M. (2001). Relations between the neural bases of dynamic auditory processing and phonological processing: evidence from fMRI. J. Cogn. Neurosci. 13, 687–698 10.1162/089892901750363235 11506664

[B122] Price C. Crinion J. MacSweeney M. (2011). A generative model of speech production in Broca's and Wernicke's areas. Front. Psychol. 2:237 10.3389/fpsyg.2011.00237 21954392 PMC3174393

[B123] Price C. J. (2012). A review and synthesis of the first 20 years of PET and fMRI studies of heard speech, spoken language and reading. Neuroimage 62, 816–847 10.1016/j.neuroimage.2012.04.062 22584224 PMC3398395

[B123a] Rao R. P. N. Ballard D. H. (1999). Predictive coding in the visual cortex: a functional interpretation of some extra-classical receptive-field effects. Nat. Neurosci. 2, 79–87 10195184 10.1038/4580

[B124] Rauschecker J. (2011). An expanded role for the dorsal auditory pathway in sensorimotor control and integration. Hear. Res. 271, 16–25 10.1016/j.heares.2010.09.001 20850511 PMC3021714

[B125] Rauschecker J. (2012). Ventral and dorsal streams in the evolution of speech and language. Front. Evol. Neurosci. 4:7 10.3389/fnevo.2012.00007 22615693 PMC3351753

[B126] Rauschecker J. Scott S. (2009). Maps and streams in the auditory cortex: nonhuman primates illuminate human speech processing. Nat. Neurosci. 12, 718–724 10.1038/nn.2331 19471271 PMC2846110

[B127] Rauschecker J. Tian B. (2000). Mechanisms and streams for processing of what and where in auditory cortex. Proc. Natl. Acad. Sci. 97, 11800–11806 10.1073/pnas.97.22.11800 11050212 PMC34352

[B128] Ravizza S. (2003). Dissociating the performance of cortical and subcortical patients on phonemic tasks. Brain Cogn. 53, 301–310 10.1016/S0278-2626(03)00131-3 14607169

[B129] Redding G. M. Wallace B. (1996). Adaptive spatial alignment and strategic perceptual-motor control. J. Exp. Psychol. Hum. Percept. Perform. 22, 379–394 10.1037//0096-1523.22.2.379 8934851

[B130] Roth M. J. Synofzik M. Lindner A. (2013). The cerebellum optimizes perceptual predictions about external sensory events. Curr. Biol. 23, 930–935 10.1016/j.cub.2013.04.027 23664970

[B131] Rothermich K. Kotz S. (2013). Predictions in speech comprehension: fMRI evidence on the meter-semantic interace. Neuroimage 70, 89–100 10.1016/j.neuroimage.2012.12.013 23291188

[B132] Rumelhart D. Hinton G. William R. (1986). Learning representations by back-propagating errors. Nature 23, 533–536 10.1038/323533a0

[B133] Samuel A. Kraljic T. (2009). Perceptual learning for speech. Atten. Percept. Psychophys. 71, 1207–1218 10.3758/APP.71.6.1207 19633336

[B134] Samuel A. G. (2011). Speech perception. Annu. Rev. Psychol. 62, 49–72 10.1146/annurev.psych.121208.131643 20809789

[B135] Schlerf J. Ivry R. Diedrichsen J. (2012). Encoding of sensory prediction errors in the human cerebellum. J. Neurosci. 32, 5913–5922 10.1523/JNEUROSCI.4504-11.2012 22492047 PMC4332713

[B136] Schwab E. Nusbaum H. Pisoni D. (1985). Some effects of training on the perception of synthetic speech. Hum. Factors. 27, 395–408 2936671 10.1177/001872088502700404 PMC3499950

[B137] Scott S. (2012). The neurobiology of speech perception and production-Can functional imaging tell us anything we did not already know? J. Commun. Sci. Disord. 45, 419–425 10.1016/j.jcomdis.2012.06.007 22840926

[B138] Scott S. K. Wise R. J. S. (2004). The functional neuroanatomy of prelexical processing in speech perception. Cognition 92, 13–45 10.1016/j.cognition.2002.12.002 15037125

[B139] Sebastian-Galles N. Dupoux E. Costa A. Mehler J. (2000). Adaptation to time-compressed speech: phonological determinants. Percept. Psychophys. 62, 834–842 10.3758/BF03206926 10883588

[B140] Seghier M. Fagan E. Price C. (2010). Functional subdivisions in the left angular gyrus where the semnatic system meets and diverges from the default network. J. Neurosci. 30, 16809–16817 10.1523/JNEUROSCI.3377-10.2010 21159952 PMC3105816

[B140a] Shadmehr R. Smith M. Krakauer J. (2010). Error correction, sensory prediction, and adaptation in motor control. Ann. Rev. Neurosci. 33, 89–108 10.1146/annurev-neuro-060909-153135 20367317

[B141] Shiller D. M. Sato M. Gracco V. L. Baum S. R. (2009). Perceptual recalibration of speech sounds following speech motor learning. J. Acoust. Soc. Am. 125, 1103–1113 10.1121/1.3058638 19206885

[B142] Skipper J. Goldin-Meadown S. Nusbaum H. Small S. (2009). Gestures orchestrate brain networks for language understanding. Curr. Biol. 19, 661–667 10.1016/j.cub.2009.02.051 19327997 PMC3767135

[B143] Sohoglu E. Peelle J. Carlyon R. Davis M. H. (2012). Predictive top-down integration of prior knowledge during speech perception. J. Neurosci. 32, 8443–8453 10.1523/JNEUROSCI.5069-11.2012 22723684 PMC6620994

[B144] Song J. Skoe E. Banai K. Kraus N. (2011). Perception of speech in noise: neural correlates. J. Cogn. Neurosci. 23, 2268–2279 10.1162/jocn.2010.21556 20681749 PMC3253852

[B145] Song J. H. Banai K. Kraus N. (2008). Brainstem timing deficits in children with learning impairment may result from corticofugal origins. Audiol. Neurotol. 13, 335–344 10.1159/000132689 18493120

[B146] Song J. H. Skoe E. Banai K. Kraus N. (2012). Training to improve hearing speech in noise: biological mechanisms. Cereb. Cortex 22, 1180–1190 10.1093/cercor/bhr196 21799207 PMC3450924

[B147] Spratling M. W. (2008). Reconciling predictive coding and biased competition models of cortical function. Front. Comput. Neurosci. 2:4 10.3389/neuro.10.004.2008 18978957 PMC2576514

[B148] Stoodley C. Schmahmann J. (2009). Functional topogaphy in the human cerebellum: a meta-analysis of neuroimaging studies. Neuroimage 44, 489–501 10.1016/j.neuroimage.2008.08.039 18835452

[B149] Stoodley C. J. Valera E. M. Schmahmann J. D. (2012). Functional topography of the cerebellum for motor and cognitive tasks: an fMRI study. Neuroimage 59, 1560–1570 10.1016/j.neuroimage.2011.08.065 21907811 PMC3230671

[B150] Strick P. Dum R. Fiez J. (2009). Cerebellum and nonmotor function. Annu. Rev. Neurosci. 32, 413–434 10.1146/annurev.neuro.31.060407.125606 19555291

[B151] Szenkovitz G. Peelle J. Norris D. Davis M. (2012). Individual differences in premotor and motor recruitment during speech perception. Neuropsychologia 50, 1380–1392 10.1016/j.neuropsychologia.2012.02.023 22521874

[B152] Thach W. T. (1998). What is the role of the cerebellum in motor learning and cognition. Trends Cogn. Sci. (Regul. Ed) 2, 331–337 10.1016/S1364-6613(98)01223-6 21227229

[B153] Tian X. Poeppel D. (2010). Mental imagery of speech and movement implicates the dynamics of internal forward models. Front. Psychol. 1:166 10.3389/fpsyg.2010.00166 21897822 PMC3158430

[B153a] Tian X. Poeppel D. (2013). The effect of imagination on stimulation: the functional specificity of efference copies in speech processing. J. Cogn. Neurosci. 25, 1020–1036 10.1162/jocn_a_00381 23469885

[B154] Tricomi E. Delgado M. McCandliss B. McClelland J. Fiez J. (2006). Performance feedback drives caudate activation in a phonological learning task. J. Cogn. Neurosci. 18, 1029–1043 10.1162/jocn.2006.18.6.1029 16839308

[B155] Turken A. U. Dronkers N. F. (2011). The neural architecture of the language comprehension network: converging evidence from lesion and connectivity analyses. Front. Syst. Neurosci. 5:1 10.3389/fnsys.2011.00001 21347218 PMC3039157

[B156] Ungerleider L. Haxby J. (1994). What and where in the human brain. Curr. Opin. Neurobiol. 4, 157–165 10.1016/0959-4388(94)90066-3 8038571

[B157] Villacorta V. Perkell J. Guenther F. (2007). Sensorimotor adaptation to feedback perturbations of vowel acoustics and its relation to perception. J. Acoust. Soc. Am. 22, 2306–2319 10.1121/1.2773966 17902866

[B158] von Kriegstein K. Dogan O. Gruterm M. Giraud A. Kell C. Gruter T. (2008). Simulation of talking faces in the human brain improves auditory speech recognition. Proc. Natl. Acad. Sci. U.S.A. 105, 6747–6752 10.1073/pnas.0710826105 18436648 PMC2365564

[B159] Vroomen J. Baart M. (2012). Phonetic recalibration in audiovisual speech, in The Neural Bases of Multisensory Processes, eds Murray M. Wallace M. (Boca Raton, FL: CRC Press), 254–259 22593864

[B160] Vroomen J. van Linden S. Degelder B. Bertelson P. (2007). Visual recalibration and selective adaptation in auditory-visual speech perception: contrasting build-up courses. Neuropsychologia 45, 572–577 10.1016/j.neuropsychologia.2006.01.031 16530233

[B161] Wild C. J. Davis M. H. Johnsrude I. S. (2012). Human auditory cortex is sensitive to the perceived clarity of speech. Neuroimage 60, 1490–1502 10.1016/j.neuroimage.2012.01.035 22248574

[B162] Wild C. J. Yusuf A. Wilson D. E. Peelle J. E. Davis M. H. Johnsrude I. S. (2012b). Effortful listening: the processing of degraded speech depends critically on attention. J. Neurosci. 32, 14010–14021 10.1523/JNEUROSCI.1528-12.2012 23035108 PMC6704770

[B163] Wolpert D. Diedrichsen J. Flanagan J. (2011). Principles of sensorimotor learning. Nat. Rev. Neurosci. 12, 739–751 10.1038/nrn3112 22033537

[B164] Wolpert D. M. Miall R. C. Kawato M. (1998). Internal models in the cerebellum. Trends Cogn. Sci. 2, 338–346 10.1016/S1364-6613(98)01221-2 21227230

[B165] Yamamoto K. Hoffman D. S. Strick P. L. (2006). Rapid and long-lasting plasticity of input-output mapping. J. Neurophysiol. 96, 2797–2801 10.1152/jn.00209.2006 16928799

[B166] Yu K. Xu W. Gong Y. (2008). Deep learning with kernel regularization for visual recognition, in Advances in Neural Information Processing Systems, (Cupertino, CA), 1889–1896

[B167] Zekveld A. Rudner M. Johnsrude I. Eslenfeld D. Ronnberg J. (2012). Behavioral and fMRI evidence that cognitive ability modulates the effect of semantic context on speech intelligibility. Brain Lang. (Baltim) 122, 103–113 10.1016/j.bandl.2012.05.006 22728131

[B168] Zheng Z. Z. Vicente-Grabovetsky A. MacDonald E. N. Munhall K. G. Cusack R. Johnsrude I. S. (2013). Multivoxel patterns reveal functionally differentiated networks underlying auditory feedback processing of speech. J. Neurosci. 33, 4339–4348 10.1523/JNEUROSCI.6319-11.2013 23467350 PMC3673229

